# Role of Akt-independent mTORC1 and GSK3β signaling in sublethal NMDA-induced injury and the recovery of neuronal electrophysiology and survival

**DOI:** 10.1038/s41598-017-01826-w

**Published:** 2017-05-08

**Authors:** Przemyslaw Swiatkowski, Ina Nikolaeva, Gaurav Kumar, Avery Zucco, Barbara F. Akum, Mihir V. Patel, Gabriella D’Arcangelo, Bonnie L. Firestein

**Affiliations:** 1 Department of Cell Biology and Neuroscience, Rutgers University, 604 Allison Road, Piscataway, New Jersey 08854-8082 USA; 2 Graduate Program in Molecular Biosciences, Rutgers University, 604 Allison Road, Piscataway, New Jersey 08854-8082 USA; 3 Graduate Program in Neurosciences, Rutgers University, 604 Allison Road, Piscataway, New Jersey 08854-8082 USA

## Abstract

Glutamate-induced excitotoxicity, mediated by overstimulation of *N*-methyl-*D*-aspartate (NMDA) receptors, is a mechanism that causes secondary damage to neurons. The early phase of injury causes loss of dendritic spines and changes to synaptic activity. The phosphatidylinositol-4,5-bisphosphate 3-kinase/Akt/ mammalian target of rapamycin (PI3K/Akt/mTOR) pathway has been implicated in the modulation and regulation of synaptic strength, activity, maturation, and axonal regeneration. The present study focuses on the physiology and survival of neurons following manipulation of Akt and several downstream targets, such as GSK3β, FOXO1, and mTORC1, prior to NMDA-induced injury. Our analysis reveals that exposure to sublethal levels of NMDA does not alter phosphorylation of Akt, S6, and GSK3β at two and twenty four hours following injury. Electrophysiological recordings show that NMDA-induced injury causes a significant decrease in spontaneous excitatory postsynaptic currents at both two and twenty four hours, and this phenotype can be prevented by inhibiting mTORC1 or GSK3β, but not Akt. Additionally, inhibition of mTORC1 or GSK3β promotes neuronal survival following NMDA-induced injury. Thus, NMDA-induced excitotoxicity involves a mechanism that requires the permissive activity of mTORC1 and GSK3β, demonstrating the importance of these kinases in the neuronal response to injury.

## Introduction

Brain trauma, caused by either mechanical injury or oxygen deprivation, leads to the stimulation or suppression of a large number of molecular signaling cascades within the brain and overall disruption of neuronal electrophysiology^1–3^. Following the initial physical damage, an almost immediate release of excitotoxic levels of amino acids, such as glutamate and aspartate, occurs in the surrounding tissues, exacerbating cell death^4^. Glutamate-induced toxicity is a common mechanism of secondary injury in both neurodegenerative disorders and neuronal injury, such as traumatic brain injury, stroke, and epilepsy^5–9^. Glutamate receptor blockers have failed to improve recovery from the secondary damage in human clinical trials, leaving researchers to examine other pathways affected by injuries.

One pathway that undergoes a dramatic increase in signaling activity after injury is the PI3K/Akt/mTORC1 pathway^10–12^ and has become a focus for drug development for the treatment of central nervous system injuries^13^. The PI3K/Akt/mTOR pathway plays an essential role in cellular growth and repair of neurons and is susceptible to modulation through glutamate receptor-mediated signaling. Excitatory levels of amino acids stimulate pathway activity; however, excitotoxic levels of amino acids lead to pathway suppression^14–16^. While many studies have focused on teasing out the role of the PI3K/Akt/mTOR pathway in damage and repair, the results of these experiments have yielded conflicting results on whether activation of PI3K/Akt/mTOR signaling is beneficial or detrimental for neuronal and functional recovery^17, 18^. Additional research at the cellular level is needed to discern the molecular and functional roles of PI3K/Akt/mTOR pathway signaling following injury.

At the cellular level, dendrites undergo morphological changes after exposure to excitotoxic levels of glutamate^19–22^. These alterations, including spine retraction and varicosity formation, also occur *in vivo* after injury^23–28^. Not much is known about the mechanisms underlying spine retraction and recovery; however, actin is thought to contribute to spine loss^29, 30^ but not to spine recovery^31^. Since actin has been linked to activation of the PI3K/Akt/mTOR pathway in local cue-induced axonal protein synthesis^32^, it is of interest to study how this pathway regulates synaptic function after injury.

**Figure 1:**
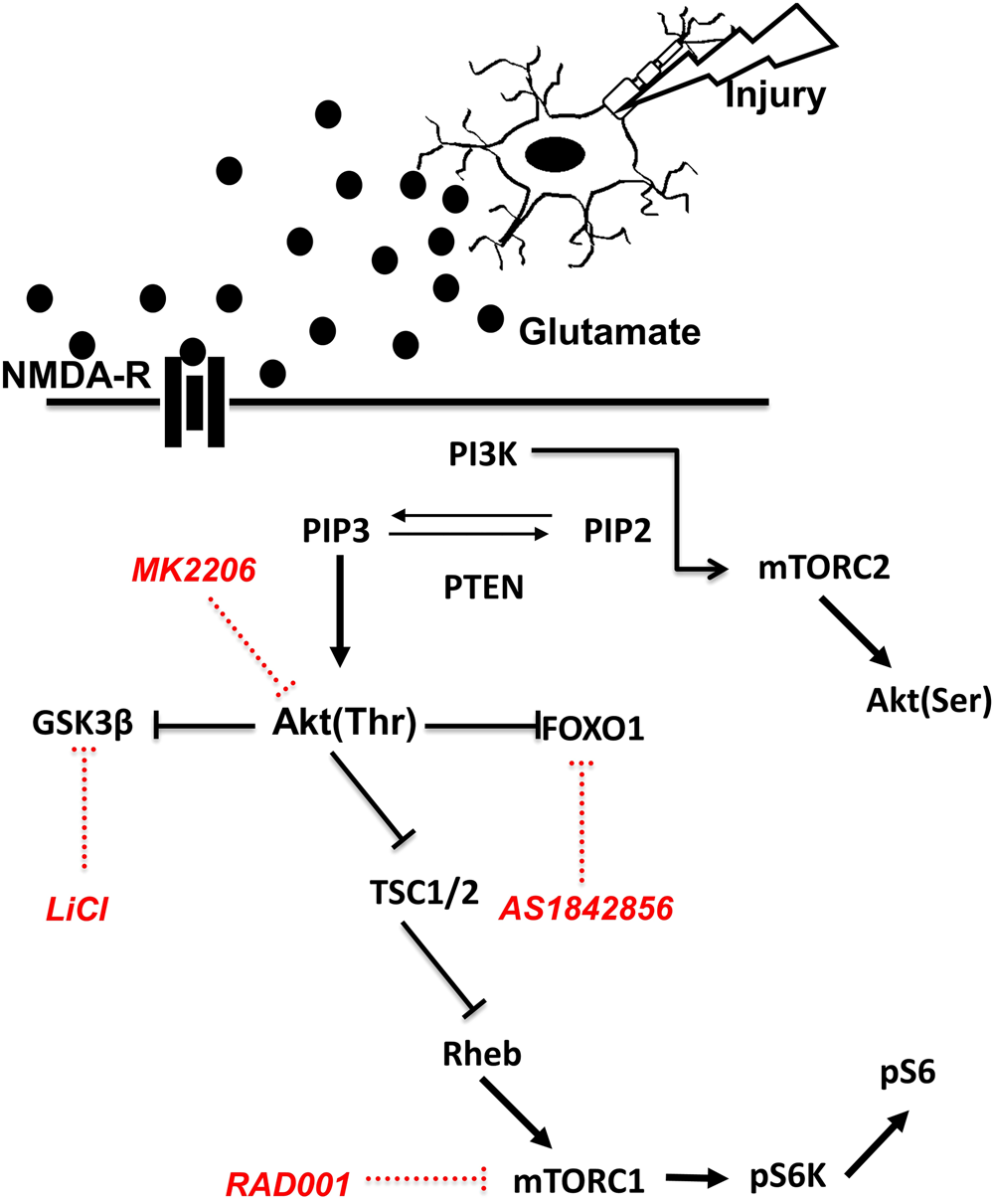
Schematic model of the PI3K/Akt/mTOR signaling pathway and pharmacological compounds used to modulate its activity. PI3K activity causes the conversion of PIP2 to PIP3, which then leads to the activation of Akt via PDK1-mediated phosphorylation on threonine 308. Additional mTORC2-dependent phosphorylation on serine 473 contributes to full Akt kinase activity. Phosphorylated Akt subsequently targets GSK3β, FOXO1, and TSC1/2 for inhibition by phosphorylation of specific residues. Suppression of TSC1/2 leads to activation of Rheb, mTORC1, and downstream targets. MK2206 inhibits Akt kinase activity, RAD001 inhibits mTORC1 activity, LiCl inhibits GSK3β activity, and AS1842856 inhibits FOXO1 function.

To study how glutamate receptor-mediated injury affects electrophysiological properties and neuronal cell survival, cultured rat cortical neurons were exposed to a sublethal concentration of NMDA. This treatment models secondary damage caused by mechanical, ischemic, or neurodegenerative injury. To test whether the PI3K/Akt/mTOR pathway plays a role in the response to NMDA, neurons were exposed to specific pharmacological agonists and inhibitors, some of which are currently in clinical trials for other diseases, such as certain cancers^33–35^, involving aberrant activity of this signaling pathway (Fig. 1). In the present study, we find that exposure of cultured neurons to sublethal levels of NMDA does not induce activation or suppression of the PI3K/Akt/mTOR pathway; however, inhibition of mTORC1 and GSK3β prior to mild excitotoxic damage aids in recovery of normal electrophysiology and survival. In contrast, inhibition of Akt does not rescue excitotoxic damage, suggesting that NMDA-mediated changes to electrophysiology and survival are independent of Akt activity, and rather, depend on selective basal activity of mTORC1 and GSK3β kinases. Together, these data demonstrate the importance of mTORC1 and GSK3β in mediating neuronal dysfunction following excitotoxic injury.

## Results

### Inhibition of mTORC1 signaling before injury leads to preserved acute neurotransmission in injured neurons

**Figure 2:**
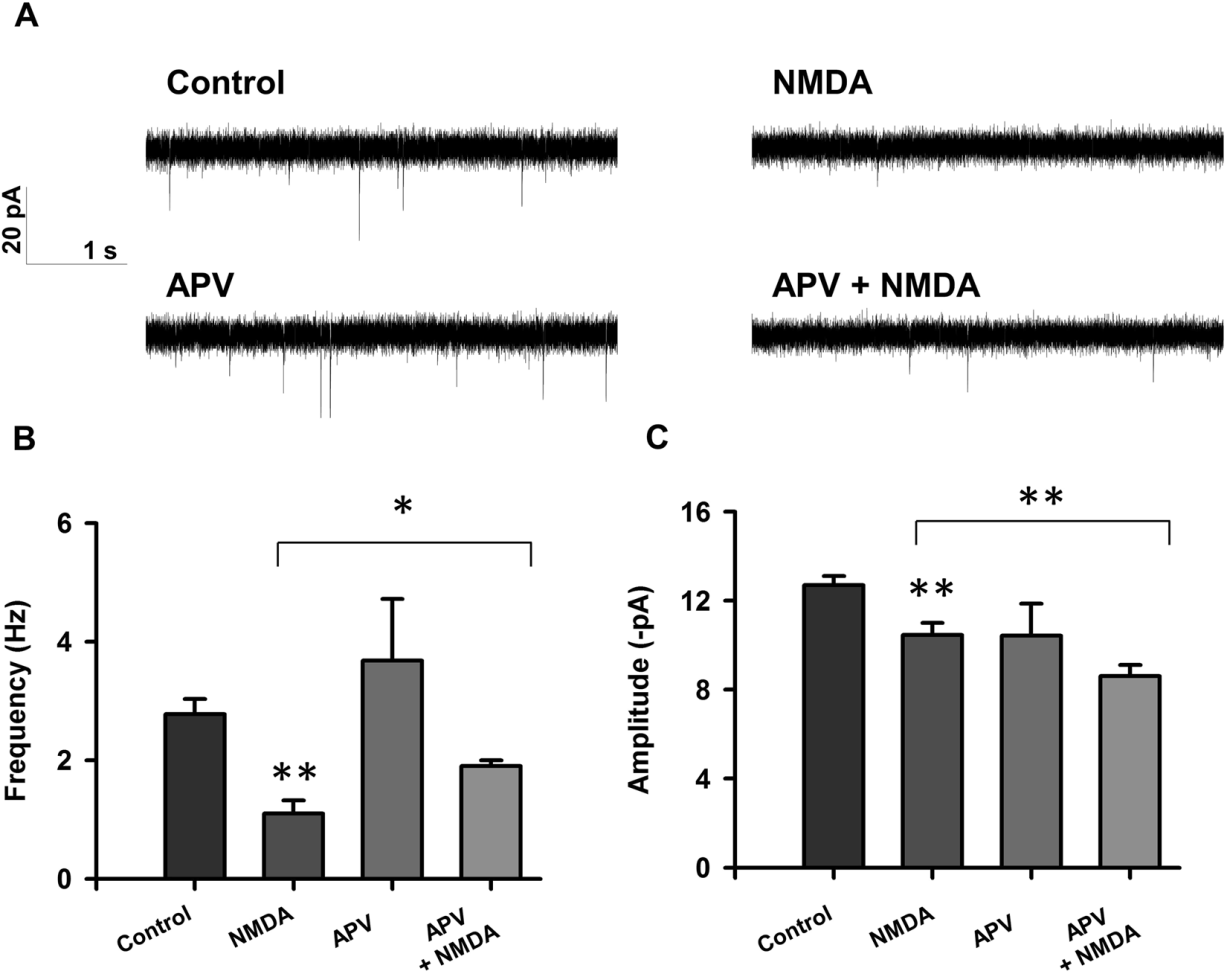
Excitotoxic injury leads to decreased sEPSC frequency and amplitude. (**A**) Representative traces of sEPSCs recorded from rat cortical neurons treated with vehicle (control; n = 74), 20 µM NMDA (n = 38), 20 µM APV (n = 6), or APV + NMDA (n = 7). (**B**,**C**) Bar graph analysis of sEPSC frequency and amplitude following 4 hour drug treatment followed by 5 minute 20 µM NMDA-induced injury and 2 hour recovery period. Data from NMDA treatment are compared to control. *p < 0.05, **p < 0.01 determined by one-way ANOVA followed by Tukey-Kramer multiple comparisons test. Error bars indicate ± SEM.

While the long term effects of excitotoxic damage on neuronal physiology are well-characterized, evidence showing acute effects is lacking. Rat cortical neuron cultures were treated on day *in vitro* (DIV) 14 with 20 μM NMDA for 5 minutes to mimic sublethal excitotoxicity as we previously described^36–38^. Electrophysiological analysis revealed that neurons treated with NMDA display a decrease in both frequency and amplitude of spontaneous excitatory postsynaptic currents (sEPSCs; Fig. 2B,C) at 2 hours following sublethal excitotoxic insult. As expected, NMDA-induced decrease in sEPSC frequency was not observed when neuronal cultures were co-treated with APV (Fig. 2B), an NMDA receptor antagonist. Interestingly, NMDA-induced decrease in amplitude was not blocked by APV, suggesting that excitotoxicity induced by synaptic NMDA receptors is, in part, responsible for the observed acute decline in neuronal activity^39, 40^.

**Figure 3:**
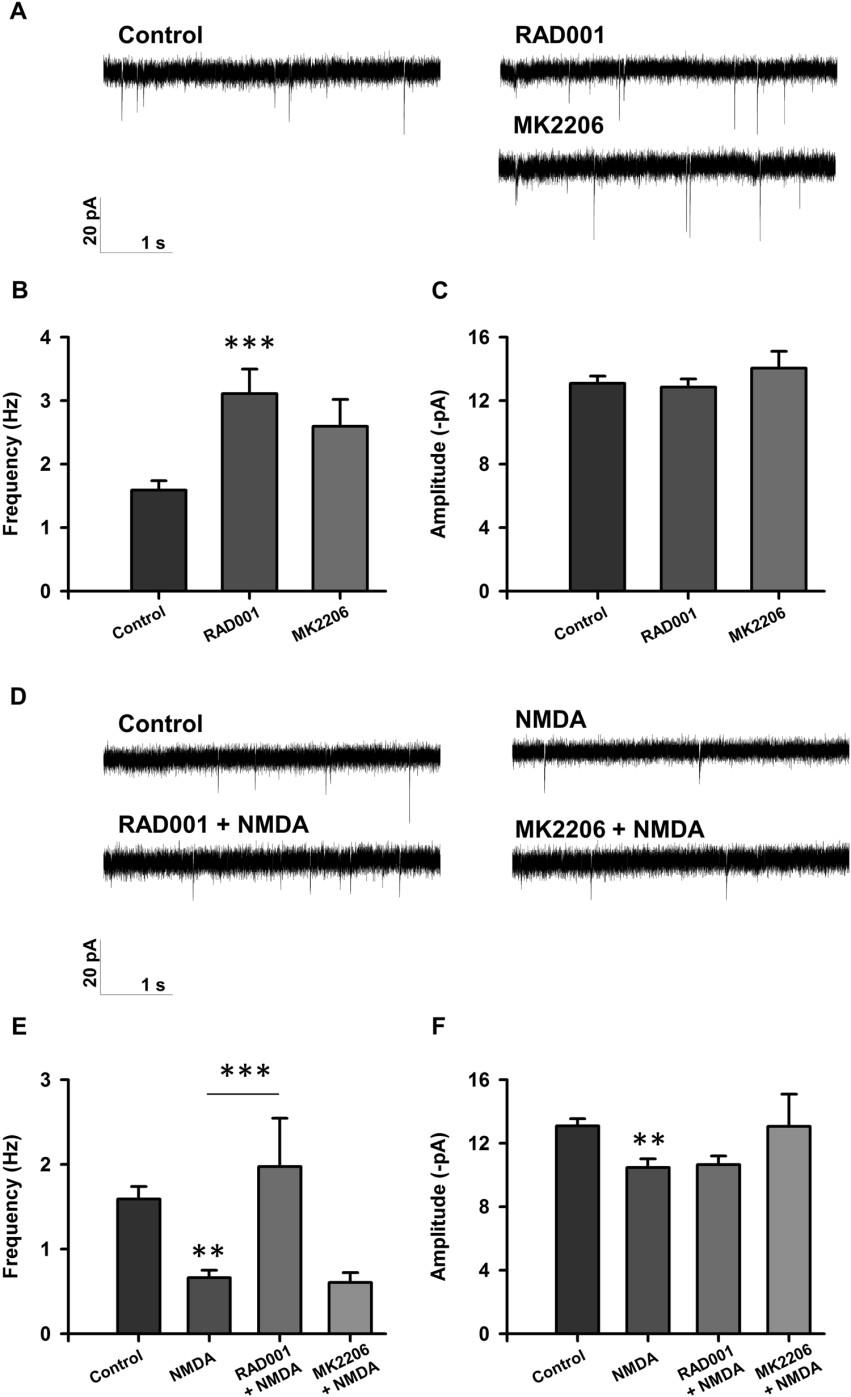
mTORC1, but not Akt, inhibition restores acute electrophysiology following injury. (**A**) Representative traces of sEPSCs recorded from rat cortical neurons treated with <0.1% DMSO (control; n = 67), 5 µM RAD001 (n = 37), or 2 µM MK2206 (n = 29). (**B**,**C**) Bar graph analysis of sEPSC frequency and amplitude following 4 hour baseline drug treatment and 2 hour recovery period. (**D**) Representative traces of sEPSCs recorded from rat cortical neurons treated with <0.1% DMSO (control; n = 67), 20 µM NMDA (n = 35), RAD001 + NMDA (n = 16), and MK2206 + NMDA (n = 13). (**E**,**F**) Bar graph analysis of sEPSC frequency and amplitude following 4 hour drug treatment, 5 minute 20 µM NMDA injury, and 2 hour recovery period. **p < 0.01, ***p < 0.005 determined by one-way ANOVA followed by Tukey-Kramer multiple comparisons test. Error bars indicate ± SEM.

To investigate the role of mTORC1 signaling on the disruption of neuronal activity in our injury paradigm, we recorded sEPSCs from control or injured neurons exposed to RAD001, an inhibitor of mTORC1 (Fig. 1). Cultures were treated with 5 μM RAD001 for four hours, followed by a five minute treatment with 20 µM NMDA to induce injury, and a two hour recovery period. We found that treatment with RAD001 alone increased the baseline frequency of sEPSCs (Fig. 3A,B). In our injury paradigm, RAD001 prevented NMDA-induced decreases in sEPSC frequency, suggesting that mTORC1 activity is detrimental for neuronal electrophysiology post-NMDA-induced injury (Fig. 3D–F). In addition, it should be noted that the apparent prevention observed with RAD001 on the effects of NDMA treatment might be ascribed to the effect of RAD001 on basal sEPSCs, consistent with a permissive role for mTORC1 in NMDA-induced changes to frequency.

We then asked whether other proteins in the PI3K/Akt/mTOR signaling pathway play a role in electrophysiological changes induced by NMDA-induced excitotoxicity. First, to demonstrate the effect of altering Akt activity on neuronal electrophysiology *in vitro*, we treated our cultures with 2 μM MK2206, an Akt inhibitor (Fig. 1), and recorded from these cultures. Treatment did not affect baseline amplitude or frequency of sEPSCs (Fig. 3A–C). We then subsequently subjected cultures treated with MK2206 to NMDA-induced injury. As described above, NMDA-induced excitotoxic damage led to a decrease in frequency and amplitude of sEPSCs two hours post-injury; however, treatment with MK2206 had no effect on sEPSC frequency and amplitude (Fig. 3D–F), suggesting that Akt activity does not play a role in the disruption of electrophysiological function due to NMDA-mediated injury. Taken together, these data suggest that mTORC1, but not Akt, activity in excitotoxic conditions mediates deleterious effects and that inhibition of mTORC1 partially prevents changes in neuronal electrophysiology.

### mTORC1, but not Akt, inhibition is beneficial for neuronal function 24 h after injury

**Figure 4:**
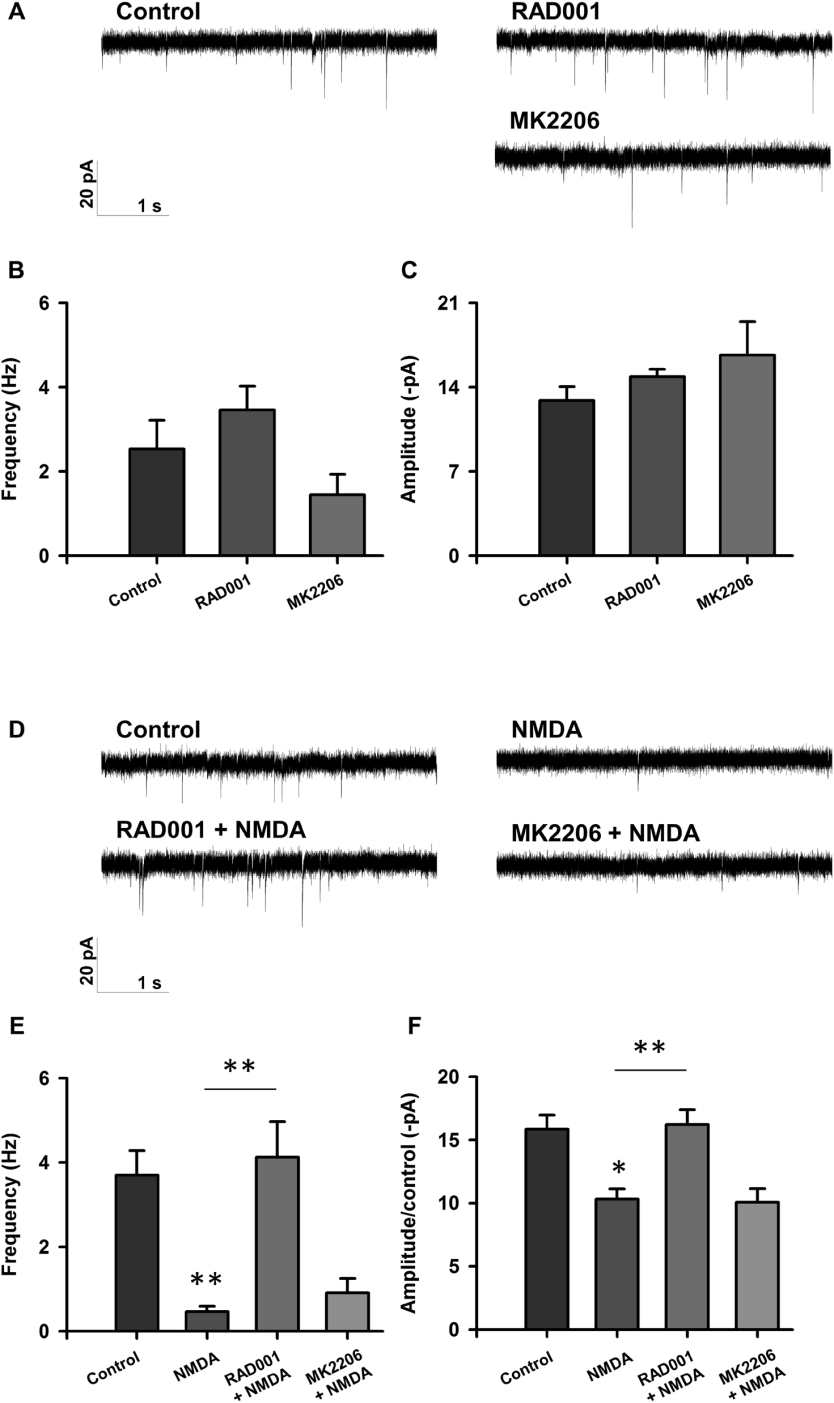
Inhibition of mTORC1, but not Akt, restores electrophysiology 24 hours following injury. (**A**) Representative traces of sEPSCs recorded from rat cortical neurons treated with <0.1% DMSO (control; n = 16), 5 µM RAD001 (n = 7), 2 µM MK2206 (n = 7). (**B**,**C**) Bar graph analysis of sEPSC frequency and amplitude following 4 hour baseline drug treatment and 24 hour recovery period. (**D**) Representative traces of sEPSCs recorded from rat cortical neurons treated with <0.1% DMSO (control; n = 29), 20 µM NMDA (n = 14), RAD001 + NMDA (n = 14), and MK2206 + NMDA (n = 15). (**E**,**F**) Bar graph analysis of sEPSC frequency and amplitude following 4 hour drug treatment, 5 minute 20 µM NMDA injury, and 24 hour recovery period. *p < 0.05, **p < 0.01 determined by one-way ANOVA followed by Tukey-Kramer multiple comparisons test. Error bars indicate ± SEM.

Our electrophysiology data recorded from neurons shortly following injury demonstrate that inhibition of mTORC1 aids in preventing alterations in neuronal electrophysiology following excitotoxic injury. To address whether the effects of this manipulation confer any long-term effects on sEPSC frequency or amplitude, as this is clinically relevant, we performed our experiments 24 hours following drug treatment and NMDA-induced injury. Both frequency and amplitude of sEPSCs were significantly decreased 24 hours after injury (Fig. 4D,E), suggesting long-term detrimental effects of excitotoxic damage on neuronal electrophysiology. At this time point, treatment with RAD001 alone resulted in no changes to baseline electrophysiology (Fig. 4A–C). However, treatment with RAD001 prevented NMDA-induced decreases in both sEPSC frequency and amplitude, whereas treatment with MK2206 had no effect (Fig. 4D–F). Taken together, these data suggest that NMDA-mediated excitotoxicity induces long-term damage to neuronal electrophysiology and that mTORC1 inhibition improves neurotransmission both acutely (two hours) and longer-term (24 hours) following injury. Akt inhibition, on the other hand, is not effective in preserving normal electrophysiology after injury.

### The role of FOXO1 and GSK3β in recovery of neuronal electrophysiology following NMDA-induced injury

Our data presented above suggest that Akt does not play a major role in mediating the effects resulting from NMDA-induced excitotoxicity; however, one of its targets (mTORC1) does. To explore the role of other signaling molecules downstream of Akt, we investigated FOXO1, a transcription factor critical for cell survival and metabolism^41^, and GSK3β, a ubiquitous enzyme implicated in glycogen metabolism and cell survival^42^. Both FOXO1 and GSK3β are phosphorylated and are functionally inhibited by Akt (Fig. 1). Additionally, FOXO1 is activated in a stress response leading to inhibition of mTORC1 and activation of Akt in order to preserve energy homeostasis (Fig. 1)^43–45^.

**Figure 5:**
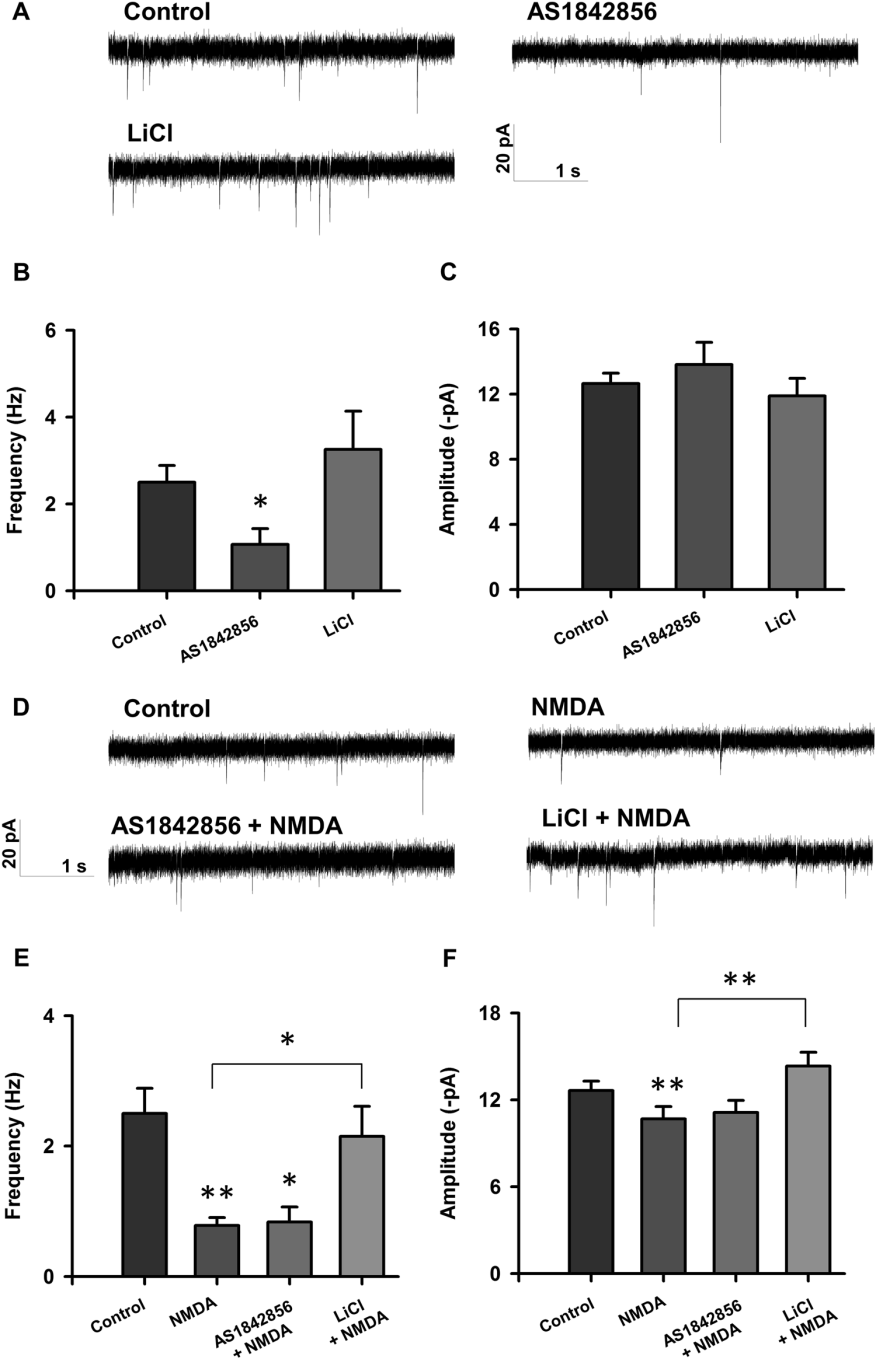
Inhibition of GSK3β, but not FOXO1, results in improved electrophysiology 2 hours following injury. (**A**) Representative traces of sEPSCs recorded from rat cortical neurons treated with <0.1% DMSO (control; n = 34), 1 µM AS1842856 (n = 15), 10 mM LiCl (n = 16). (**B**,**C**) Bar graph analysis of sEPSC frequency and amplitude following 4 hour baseline drug treatment and 2 hour recovery period. (**D**) Representative traces of sEPSCs recorded from rat cortical neurons treated with <0.1% DMSO (control; n = 34), 20 µM NMDA (n = 22), AS1842856 + NMDA (n = 12), LiCl + NMDA (n = 18). (**E**,**F**) Bar graph analysis of sEPSC frequency and amplitude following 4 hour drug treatment, 5 minute 20 µM NMDA-induced injury, and two hour recovery period. *p < 0.05, **p < 0.01 determined by one-way ANOVA followed by Tukey-Kramer multiple comparisons test. Error bars indicate ± SEM.

**Figure 6:**
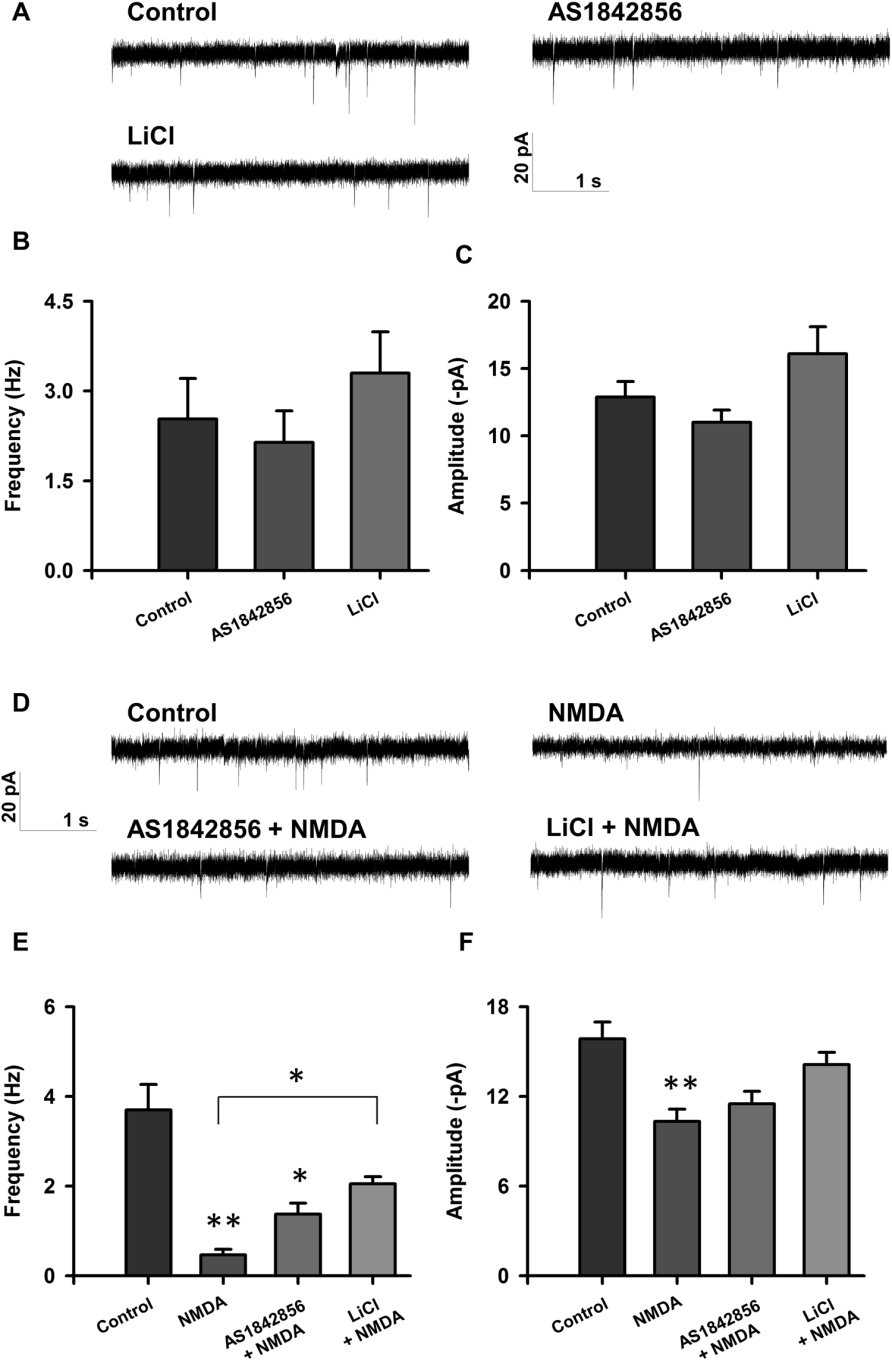
Inhibition of GSK3β results in recovery of electrophysiology 24 hours following NMDA-induced injury. (**A**) Representative traces of sEPSCs recorded from rat cortical neurons treated with < 0.1% DMSO (control; n = 16), 1 µM AS1842856 (n = 16), 10 mM LiCl (n = 10). (**B**,**C**) Bar graph analysis of sEPSC frequency and amplitude following 4 hour baseline drug treatment and 24 hour recovery period. (**D**) Representative traces of sEPSCs recorded from rat cortical neurons treated with < 0.1% DMSO (control; n = 29), 20 µM NMDA (n = 14), AS1842856 + NMDA (n = 9), LiCl + NMDA (n = 27). (**E**,**F**) Bar graph analysis of sEPSC frequency and amplitude following 4 hour drug treatment, 5 minute 20 µM NMDA-induced injury, and 24 hour recovery period. *p < 0.05, **p < 0.01 determined by one-way ANOVA followed by Tukey-Kramer multiple comparisons test. Error bars indicate ± SEM.

To investigate how manipulation of these Akt downstream targets affects neuronal electrophysiology, we treated neurons with AS1842856, a FOXO1 inhibitor (Fig. 1), for 24 hours or LiCl, a GSK3 inhibitor (Fig. 1), for four hours prior to injury. While treatment with AS1842856 resulted in decreased baseline sEPSC frequency two hours following treatment (Fig. 5A–C), but not 24 hours after treatment (Fig. 6A–C), inhibition of FOXO1 had no effect on NMDA-induced electrophysiological changes by two or 24 hours post-injury (Figs 5D–F and 6D–F). In contrast, inhibition of GSK3 with LiCl had no effect on baseline neurotransmission (Figs 5A–C and 6A–C) but prevented NMDA-induced changes to sEPSCs to control, uninjured amplitude and frequency at two hours, and partial recovery at 24 hours following injury (Figs 5D–F and 6D–F). The partial recovery at 24 hours may represent the fact that inhibition of GSK3 early is not sufficient to maintain full recovery of sEPSCs at this time point.

### The roles of mTOR pathway components in NMDA-induced changes to mEPSCs and sEPSCs are identical

Since the observed decreases in sEPSC frequency and amplitude after NMDA treatment might be attributed to changes in neuronal excitability, we recorded miniature-EPSCs (mEPSCs), which are independent of action potentials. Analysis of mEPSCs revealed that NMDA-induced changes are almost identical to the decreased frequency and partially decreased amplitude observed at 2 hours following sublethal excitotoxic insult (Supplementary Figure S1D–F). Neurons co-treated with mTORC1 and GSK3β inhibitors before injury did not show this reduction. Interestingly, treatment with either the Akt or FOXO1 inhibitor alone induced reduction of mEPSC frequency (Supplementary Figure S1A–C).

Furthermore, we quantified any changes to dendritic arborization and number of synapses resulting from NMDA-induced injury or the inhibitors used in this study, potentially providing mechanistic insight into observed changes in neuronal electrophysiology. Our data show that arborization of dendrites, which are MAP2-immunopositive, does not change in response to exposure to NMDA or inhibitors as evidenced by Sholl analysis (Supplementary Figure S2). In addition, treatment with NMDA or inhibitors does not result in changes to the number of excitatory synapses, as determined by the number of axonal synaptophysin-positive clusters apposing dendritic PSD-95 clusters per 10 µm dendrite (Supplementary Figure S3A). While these data show no effect on the number of synapses in our cultures by NMDA and inhibitors used in this study, electrophysiological data suggest that a subset of these synapses are dysfunctional.

Taken together, our data suggest that inhibition of GSK3 activity has a beneficial effect on function of cortical neurons after injury and may be a therapeutic target for managing the effects of excitotoxic damage. In addition, based on our data, GSK3 signaling is parallel to mTORC1 signaling in mediating synaptic and electrophysiological changes in response to NMDA-induced injury. Since Akt inhibition is not sufficient to suppress NMDA-induced effects, we propose that both mTORC1 and GSK3 function independently of Akt in this process.

### Sublethal levels of NMDA do not activate the PI3K/Akt/mTOR pathway

**Figure 7:**
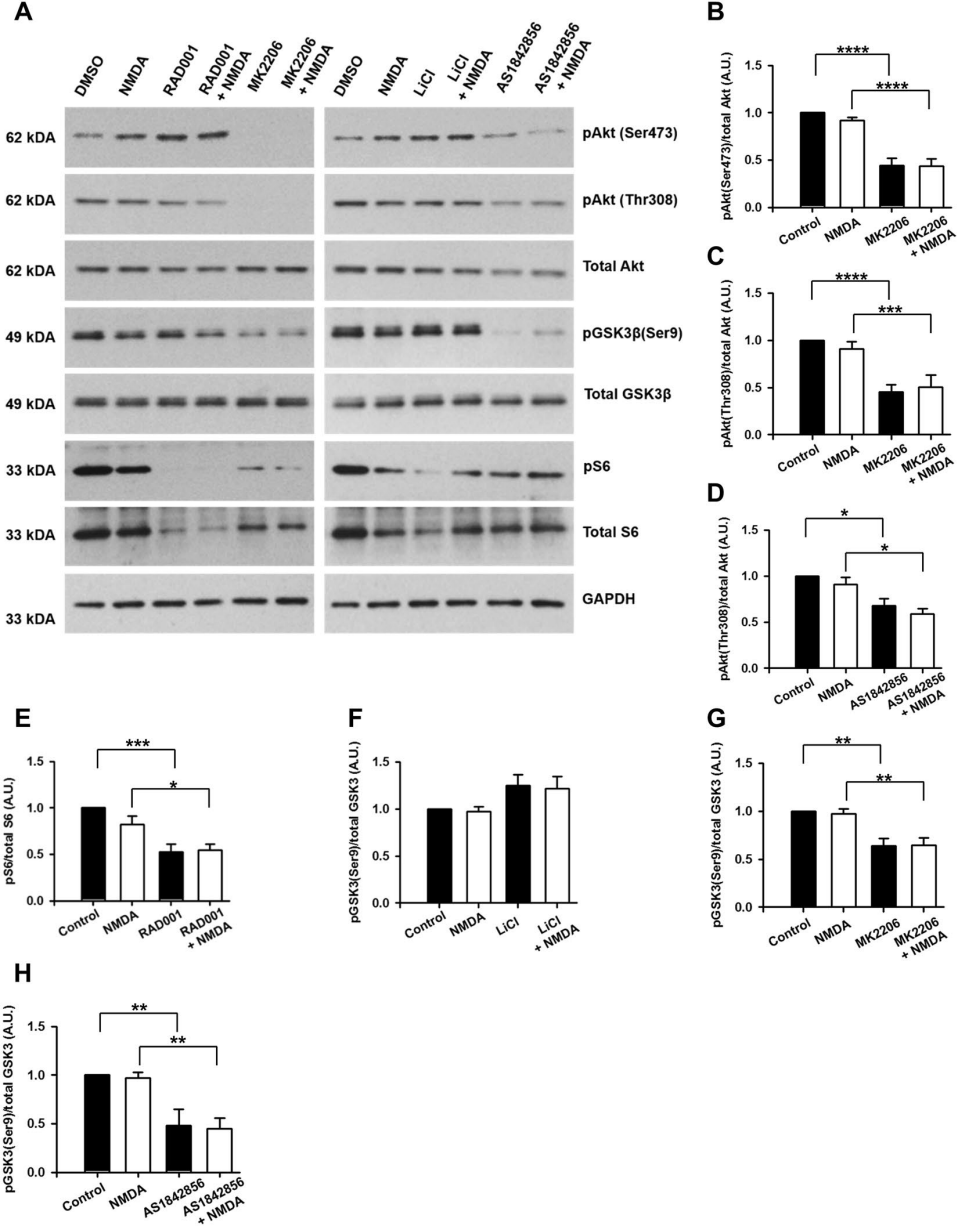
Sublethal excitotoxic injury does not induce phosphorylation of downstream targets of Akt at 2 hours after injury. (**A**) Representative Western blot bands showing phosphorylation of threonine 308 in Akt (pAkt(Thr308)) and serine 473 in Akt (pAkt(Ser473)) and total Akt, phosphorylation of S6 (pS6) and total S6, phosphorylation of serine 9 in GSK3β (pGSK3β(Ser9)) and total GSK3β, from cortical neuron cultures treated with 0.1% DMSO (control), NMDA (20 µM), RAD001 (5 µM), MK2206 (2 µM), LiCl (10 mM), and AS18425856 (1 µM) and allowed to recover for two hours. (**B**–**H**) Quantitative analysis of band intensity shows MK2206-induced inhibition of phosphorylation of Akt and GSK3β, RAD001-induced inhibition of phosphorylation of ribosomal protein S6, LiCl induced phosphorylation of GSK3β, and AS1842856-induced inhibition of phosphorylation of Akt and GSK3β. Western blot bands are representative from six replicates of the experiment. *p < 0.05, **p < 0.01, ***p < 0.005, ****p < 0.001 determined by two-way ANOVA followed by Tukey’s multiple comparisons test. Error bars indicate ± SEM.

To investigate whether the acute effects on neuronal physiology after sublethal NMDA-mediated injury are associated with activation of the PI3K/Akt/mTOR pathway, we performed Western blot analysis on protein extracts from cultures at two (Fig. 7) and 24 hours after NMDA treatment. We found that NMDA treatment did not induce phosphorylation of Akt on threonine 308 (pAkt(Thr308)) or serine 473 (pAkt(Ser473)), ribosomal protein S6 on serine 235/236 (pS6), and GSK3β on serine 9 (pGSK3β) when compared to levels of total Akt, S6, and GSK3β (Fig. 7; n = 6). These data, in contrast to published literature, show that sublethal exposure to NMDA does not activate PI3K/Akt/mTOR pathway at two and 24 hours^46, 47^.

Since we observed a lack of activation of the PI3K/Akt/mTOR pathway by NMDA, we asked how selective modulation of the downstream targets of Akt affects different components of the PI3K/Akt/mTOR pathway and whether the data observed for mTOR and GSK3 involvement in NMDA-induced changes to electrophysiology suggest a permissive role for these effectors. We took a pharmacological approach to establish the role of individual kinases in NMDA-induced excitotoxicity. To confirm the specificity of our drug treatments in our culture conditions, we either pretreated cultures for four hours with 0.01% DMSO (as a vehicle control^36–38^), Akt inhibitor MK2206 (2 μM), mTORC1 inhibitor RAD001 (5 μM), GSK3β inhibitor LiCl (10 mM) or pretreated cultures for twenty four hours with FOXO1 inhibitor AS1842856 (1 µM) and then either induced sublethal injury with 20 μM NMDA for 5 minutes. Control cultures were treated with vehicle. Cultures were allowed to recover for 2 hours without the presence of these inhibitors, at which point, cells were lysed, and proteins were extracted for Western blot analysis. As expected, exposure of cultures to MK2206 resulted in significantly decreased levels of pAkt(Thr), pAkt(Ser), and pGSK3β (Fig. 7B,C,G), and exposure of cultures to RAD001 resulted in decreased pS6 (Fig. 7E). Furthermore, LiCl induced a modest increase in pGSK3β (p = 0.055, Fig. 7F), while exposure to AS1842854 resulted in decreased pAkt(Thr) and pGSK3β levels (Fig. 7D,H).

To determine whether the effects observed on the PI3K/Akt/mTOR signaling pathway are maintained for a longer time period after NMDA exposure, we performed similar Western blot analysis of cellular extracts from cultures allowed to recover for 24 hours post-injury. We found that at this time point, phosphorylation levels of targets of the pathway were highly variable, and thus, the activation status of the signaling kinases could not be determined (data not shown).

Taken together, our data suggest that exposure to sublethal levels of NMDA does not change the activation state of the PI3K/Akt/mTORC1 signaling pathway and that the drugs used act to inhibit their intended targets under our experimental conditions. Furthermore, our data suggest that basal activity of mTOR and GSK3β acts permissively to allow NMDA to induce damage to neurons. This permissive role is supported by data that inhibition of GSK3 activity by LiCl (Fig. 5) restores sEPSCs to control, uninjured amplitude and frequency but that GSK3β is not phosphorylated by NMDA-induced injury. Additionally, loss of some protection by LiCl after 24 hours also suggests that GSK3β may only play a permissive role within hours after injury.

### Inhibition of mTORC1 and GSK3β promotes neuronal survival after NMDA treatment

**Figure 8:**
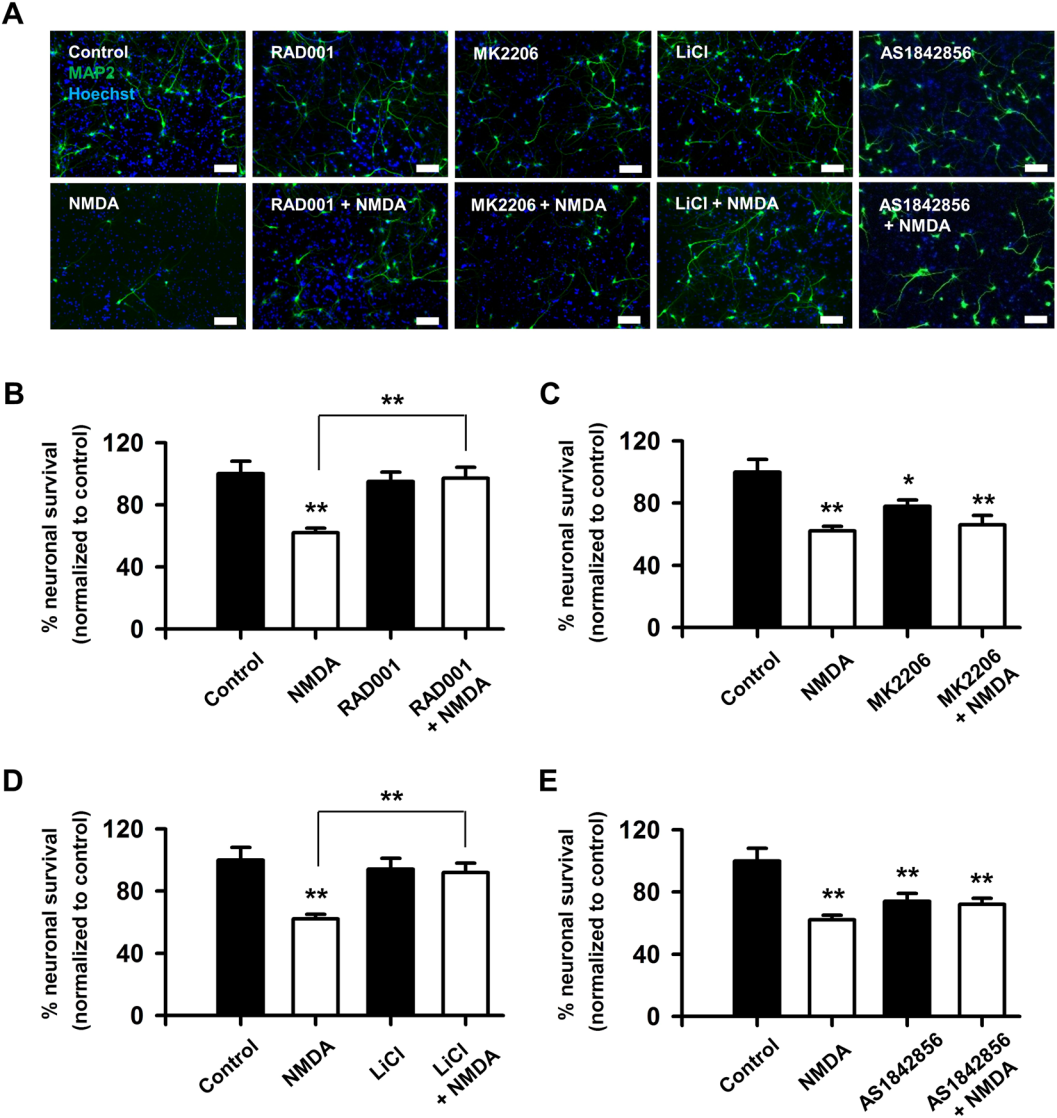
mTORC1 and GSK3β inhibitors protect neurons from NMDA-induced death. (**A**) Representative images showing neurons immunostained for the neuronal marker, MAP2, and co-stained with nuclear dye, Hoechst, after treatment with 0.1% DMSO (control), 20 µM NMDA, 5 µM RAD001, 2 µM MK2206, 10 mM LiCl, 1 µM AS1842856, with or without 20 µM NMDA. Scale bars: 100 µm. (**B**–**E**) Quantitative analysis of neuron survival expressed as percent live control neurons. RAD001 or LiCl pretreatment prevented NMDA-induced death. Data represent 38–53 samples from three separate trials. *p < 0.05, **p < 0.01 determined by one-way ANOVA followed by Tukey-Kramer multiple comparisons test. Error bars indicate ± SEM.

The data presented thus far suggest a protective effect of RAD001 and LiCl treatment on neuronal electrophysiology both acutely and 24 hours after injury. Since these inhibitors did not change the phosphorylation state of Akt (Fig. 7), we asked whether in addition to improved electrophysiology, these inhibitors improve neuronal survival after injury. Thus, we quantified the number of surviving neurons in our cultures at 24 hours after treatment with 0.01% DMSO (vehicle), RAD001 (5 µM), MK2206 (2 µM), LiCl (10 mM), AS1842856 (1 µM) and either NMDA or vehicle (as described above). Interestingly, NMDA treatment induced a 38% decrease in neuron count when compared to cultures treated with vehicle, while both RAD001 and LiCl prevented any substantial neuronal death (Fig. 8A,B,D). In contrast, both MK2206 and AS1842856 induced death of control neurons and had no effect on survival of cultures treated with NMDA (Fig. 8A,C,E).

## Discussion

Excessive release of glutamate from neurons occurs in response to tearing, stretching, or nutrient deprivation in the brain^48, 49^. High levels of extracellular glutamate lead to overactivation of receptors, and in specific, NMDA receptors^50^, on neighboring neurons and subsequent excitotoxicity^38, 49, 50^. This cellular mechanism underlies damage to neuronal networks due to injury, disease, and neurocognitive disorders. Existing treatment therapies employ NMDA antagonists to limit excitotoxic damage and aid in recovery of cellular and cognitive health of affected individuals. Unfortunately, many of the clinical trials ultimately fail, due to the importance of normal NMDA function in the brain^51^. Thus, the identification of alternative drug targets for the treatment of the damaged brain is of importance.

In the current study, we focused on the role of the Akt and its downstream signaling molecules in electrophysiological recovery and survival after injury. Glutamate release caused by excitotoxic damage has been previously associated with Akt inhibition and caspase-independent and dependent cell death^48, 52–56^. In our experimental design, we treated cultures with a sublethal concentration of NMDA (20 μM), as previously described^57^, to mimic the secondary phase of trauma, excitotoxic injury. Our data demonstrate no activation of the PI3K/Akt/mTORC1 pathway two or twenty four hours following exposure, which differs from previous reports that trauma induces this signaling pathway activation *in vivo* ^12, 41, 58^. Our data may be different since our study employs cultured neurons treated with NMDA while other studies were performed *in vivo*. Furthermore, it is possible that NMDA increases activation of mTOR and GSK3β at time points that we did not analyze. Additionally, our paradigm simulates sublethal NMDA-induced injury while more severe injuries *in vivo* may show activation of components of the mTOR signaling pathway. Regardless, our data suggest an important, yet permissive, novel role for GSK3β, and an established role for mTOR, in mediating the effects of NMDA-induced injury.

While the long-term effects of glutamate-induced excitotoxicity generally involve epileptic seizures, disruption of long-term potentiation and depression, dysregulated sEPSCs, and miniature EPSCs^48, 50, 59^, the acute effects of injury on neuronal electrophysiology are poorly characterized. In this study, we demonstrated that NMDA-induced sublethal damage causes a significant decrease in both frequency and amplitude of sEPSCs. This effect was partially recovered by APV, an NMDA antagonist, confirming the importance of NMDA receptors in mediating excitotoxic damage. Until now, the effect of PI3K/Akt/mTOR pathway manipulation on neuronal electrophysiology following injury, however, has remained largely unknown. Using the FDA approved drug RAD001, we report that inhibition of mTORC1 leads to recovery of frequency and partial recovery of amplitude of sEPSCs at two and 24 hours following injury. Additionally, RAD001 causes a significant increase in baseline activity, which in itself could be protective against upcoming injury by NMDA. Recovery is also seen upon manipulation of GSK3 but not FOXO1. GSK3β plays a role in control of several voltage-gated channels and ligand-gated receptors^60, 61^. It is important to note, however, that LiCl induces full recovery acutely, but only partial recovery 24 hours following injury (Fig. 6D–F). In light of the fact that 20 µM NMDA does not induce phosphorylation of GSK3β (Fig. 7A,F), our results suggest that GSK3β may play a permissive role in allowing NMDA to induce damage hours after injury but that inhibition of GSK3β cannot fully rescue the neurons at later time points, such as 24 hours. Inhibition of GSK3 has been implicated in internalization of AMPA and NMDA receptors, potentially leading to a decrease in the amount of intracellular Ca^2+^ and reduced excitotoxicity^62, 63^. These mechanisms may underlie the role of GSK3β in mediating neuroprotection. Here, we report that the use of the FDA approved and commonly used antipsychotic and GSK3 inhibitor, LiCl, leads to recovery of neuronal electrophysiology post-injury. We posit that this recovery is caused by either reduction of the extent of injury due to the reduced function of GSK3 in AMPA-mediated NMDA recycling from the postsynaptic density or a direct role of GSK3 in neuronal dysfunction after injury.

Changes to neuronal survival and spines have been characterized in several *in vivo* and *in vitro* models of excitotoxicity^19, 20, 22^. The PI3K/Akt/mTOR signaling cascade is a key player in dendrite and axon growth and repair^64^; however, the role of this pathway in TBI, stroke, and other neurodegenerative diseases is either controversial or largely unknown^11, 58^. Interestingly, our data demonstrate that MK2206, a potent Akt inhibitor, has no effect on neuronal survival, while RAD001 and LiCl, FDA approved mTORC1 and GSK3 inhibitors, respectively, cause a significant increase in neuronal survival after sublethal injury. Given that mTORC1 and GSK3β are both targeted by Akt, but mTORC1 is activated whereas GSK3β is inhibited by Akt-dependent phosphorylation events, these results may appear contradictory. We speculate that inhibition of both mTORC1 and GSK3 (possibly GSK3α) is beneficial following NMDA-induced injury. Our data are most consistent with a model in which both mTORC1 and GSK3 are targeted by upstream signaling molecules other than Akt to mediate injury or launch repair and survival processes. For example, mTORC1 may be activated by the Ras/MEK/ERK pathway^65, 66^, or by phospholipase D^67, 68^. mTORC1 could also be inhibited by AMP-activated protein kinase^69–71^, hypoxia-inducible REDD1 gene and the TSC1/2 complex^72^. Importantly, inhibition by REDD1 and TSC1/2 occurs in response to hypoxia^73–75^, resulting in mTORC1 activation. mTORC1 can also be activated by elevated levels of amino acids through MAP3K3, representing an additional mechanism by which NMDA may act to regulate this kinase activity^76^. Similarly, NMDA exposure could regulate GSK3 activity independently of Akt, despite the fact that GSK3β is a well-known Akt target. Although less is known about how this may occur, there are reports reflecting such a pathway. In murine sensory neurons, regeneration is mediated by Akt-independent GSK3β inactivation^77^. In human non–small-cell lung cancer cell lines, degradation of cellular FLICE-inhibitory protein by celecoxib is mediated by GSK3 but not Akt^78^, and in murine neuroblastoma cells, estradiol regulates GSK3β activity independent of Akt^79^. Furthermore, GSK3β has been extensively investigated for its role in cancer progression where it is reported to be under control of several different pathway including PI3K/Akt/mTOR, Ras/Raf/MEK/ERK, Wnt/β-catenin, Hedgehog, and Notch^80^. Thus, Akt-independent regulation of GSK3 by NMDA to alter electrophysiology and survival of neurons may represent a novel pathway by which neurons react to injury.

It is important to note that since mTORC1 appears to mediate a deleterious effect of NMDA-mediated injury, the therapeutic window for treatment with an mTORC1 inhibitor to counteract this effect is likely short. Similar to the failure of NMDA antagonists in clinical trials for traumatic brain injury, the targeting of deleterious pathways during injury requires timely inhibitor administration to prevent irreversible damage to occur. However, since suppression of GSK3 activity may be part of the recovery response of the cell to injury, the therapeutic window for stimulating repair and recovery responses may last days or even months. Therefore, pharmacological targeting of the GSK3 and mTORC1 pathways may distinct beneficial effects if initiated at different times before and after NMDA-induced injury.

Taken together, our data suggest a novel role for parallel mTORC1 and GSK3 signaling and manipulation on neuronal recovery following excitotoxic damage, a common secondary injury mechanism shared among multiple brain injuries and diseases. We show that mTORC1 and GSK3 inhibition result in recovery of both frequency and amplitude of sEPSCs two and 24 hours following injury. Since our data suggest that these pathways act in an Akt-independent manner, future studies aimed at elucidating the upstream molecular mechanisms governing neuronal recovery will provide details for precise manipulation of these signaling molecules in brain injury and disease.

## Methods

### Ethical approval

All methods using animals in this manuscript were approved by the Rutgers Institutional Animal Care and Use Committee in accordance with the Panel on Euthanasia of the AVMA.

### Primary cortical neuron culture and injury

Neuronal cultures were plated from cortices of rat embryos at 18 days gestation on glass coverslips (12 mm diameter; 176,911 cells/cm^2^ for electrophysiology and Sholl analysis, 47,244 cells/cm^2^ for immunocytochemistry) or 6 well Falcon tissue culture plates (9.6 cm^2^; 104,167 cells/cm^2^ for Western blot analysis) previously coated with poly-D-lysine (Sigma-Aldrich) in full Neurobasal medium (Life Technologies, Grand Island, NY) supplemented with B-27 (Life Technologies) and GlutaMax (Life Technologies). At day *in vitro* (DIV) 14, cultures were treated with 20 µM NMDA in 0.1% DMSO for 5 minutes, and allowed either 2 or 24 hours of recovery before electrophysiological experiments or Western blot analysis.

### Antibodies

All antibodies, with the exception of mouse anti-actin (EMD Millipore, Billerica, MA) and Akt1/2/3 antibody for immunostaining (sc-8312, Santa Cruz Biotechnology), were purchased from Cell Signaling (Danvers, MA) and were used 1:1000 dilution. Catalogue numbers for each antibody are as follows: pAkt(Thr308) (Rb, 2965), pAkt(Ser473) (Rb, 9271), total Akt (Rb, 4691), pS6(Ser235/236) (Rb, 4858), total S6 (M, 2317), pGSK3β(Ser9) (Rb, 9323), total GSK3β (Rb, 9315).

### Drugs

N-methyl-D-aspartate (NMDA) was purchased from Sigma-Aldrich (St. Louis, MO). RAD001 and MK2206 were purchased from Selleckchem (Houston, TX). AS1842856 was purchased from EMD Millipore (Billerica, MA). LiCl and dimethyl sulfoxide (DMSO) were purchased from Thermo Fisher Scientific (Waltham, MA).

### Immunocytochemistry

Neurons were grown for 14 days in culture on coverslips coated with poly-D-lysine at which point they were treated with 20 µM NMDA for 5 minutes. Cultures were fixed at 15 days in culture in 4% paraformaldehyde in PBS for 10 min, permeabilized with 0.1% Triton X-100 in PBS + 5% normal goat serum, and immunostained with mouse anti-MAP2 (1:500) from Rockland, followed by secondary antibodies conjugated to Alexa-Fluor® 488 (Invitrogen, 1:250). Nuclei were stained with Hoechst dye (1:1000). Coverslips were mounted on glass slides with Fluoromount G and then imaged. Neurons were visualized by immunofluorescence under a 10X objective on an EVOS FL microscope. Only neurons positive for MAP2 immunostaining and Hoechst staining were counted and used for statistical analysis^22, 81, 82^.

### Semi-Automated Sholl analysis

Hippocampal neurons were grown for 14 days in culture on coverslips coated with poly-D-lysine and transfected with pEGFP using Effectene (Qiagen) following the manufacturer’s instructions. Neurons were then fixed in 4% paraformaldehyde in PBS for 10 min, permeabilized with 0.1% Triton X-100 in PBS + 5% normal goat serum, and immunostained with rabbit anti-MAP2 (1:500) and mouse anti-GFP (1:500) followed by secondary antibodies conjugated to Alexa-Fluor® 555 or Alexa-Fluor® 488 (all antibodies from Rockland). Coverslips were mounted on glass slides with Fluoromount G and then imaged. Neurons were visualized by immunofluorescence under a 20X objective on an Olympus IX50 microscope with a Cooke Sensicam CCD-cooled camera, fluorescence, imaging system, and Image Pro software. The experimenter was blinded to the condition when taking images and analyzing dendrite morphology. Only GFP-positive neurons with clear neuronal morphology were examined. Next, 8-bit images were used to trace neurons using the NeuronJ plugin for ImageJ (NIH, Bethesda, MD). Tracing files were generated, and using MATLAB (Mathworks), these tracing files were subsequently converted to SWC files, and connectivity was assessed using NeuronStudio software. Sholl analysis was performed using a 6 µm ring interval starting at 9.3 µm from the soma^37^. Raw data were then exported to Excel using MATLAB and subjected to statistical analysis. Sholl analysis was performed using our Bonfire software^37, 83^, which is freely available to the scientific community.

### Synaptic Cluster analysis

Cortical neurons (47,244 cells/cm^2^) grown on glass coverslips (12 mm) for 14 days in culture were fixed in 4% paraformaldehyde in phosphate-buffered saline (PBS) for 10 min, permeabilized with 0.1% Triton X-100 in PBS + 5% normal goat serum, and immunostained with rabbit anti-synaptophysin (Zymed Laboratories; 1:500) and mouse anti-PSD-95 (Antibodies Inc; 1:500) followed by secondary antibodies conjugated with Alexa-Fluor® 488 or Alexa-Fluor® 647 (Invitrogen; 1:250). Images of dendritic segments were taken with a 60X Plan Apo oil-immersion objective (NA 1.4) using a Yokogawa CSU-10 spinning disk confocal head attached to an inverted fluorescence microscope (Olympus IX50). X-Y and Z-resolution were set as 0.067 μm-0.067 μm and 0.2 μm, respectively, to count synaptic puncta. Puncta along dendritic segments were counted from 20 μm to 80 μm from the soma for 15–30 µm of secondary or tertiary dendrite segment length. Puncta were counted for at least 20 neurons for each experimental condition, and analysis was performed with the experimenter blinded to the condition. Synapse Counter (ImageJ plug in) was used to analyze 21 apposition of synaptophysin and PSD-95 puncta^84^. Statistical analysis was performed using One-way ANOVA followed Tukey’s multiple comparisons test in Instat (Graphpad).

### Western Blot analysis

On DIV 10, neuronal cultures plated in Falcon tissue culture plates were lysed in RIPA lysis buffer (50 mM Tris (pH 7.4), 1% NP40, 0.25% sodium deoxycholate, 150 mM NaCl, 1 µM EDTA supplemented with protease and phosphatase inhibitors), and extracts were spun at 4 °C at 3000× *g* for 5–7 minutes to remove debris. Protein concentration was measured by a standard Bradford assay. Proteins (15 µg per sample) were resolved by SDS-polyacrylamide electrophoresis using 8.0% gels and transferred onto 0.2 μm nitrocellulose membrane (Bio-Rad, Hercules, CA) at 4 °C in 20% methanol Tris-Glycine buffer (25 mM Tris, 192 mM glycine, 20% v/v methanol) for 2.5 hours at a constant current (0.5 Amp). Membranes were blocked in 3% milk in TBS-T (50 mM Tris, 150 mM NaCl, pH 7.6) for 1 hour and incubated in primary antibodies overnight at 4 °C. Membranes were then incubated for 1 hour in secondary HRP-conjugated antibodies and subjected to ECL-Plus Western Blotting Detection System (Pierce/Thermo Fisher, Rockland, IL). All phosphorylated protein levels were normalized to the total amount of each protein. Quantification was performed using the band analysis function of Alpha Imager software (Protein Simple).

### Electrophysiology

Whole cell patch-clamp recordings were performed on the soma of cortical neurons. For recordings, cells were bathed in artificial cerebrospinal fluid containing (in mM): 140 NaCl, 5 KCl, 2 CaCl_2_, 2 MgCl_2_, 10 HEPES, and 10 glucose (pH 7.4 adjusted with NaOH; 290–310 mOsmol). Recording electrodes (3–5 MΩ) contained a K^+^-based internal solution composed of (in mM): 126 K-gluconate, 4 KCl, 10 HEPES, 4 ATP-Mg, 0.3 GTP-Na_2_, and 10 phosphocreatine (pH 7.2; 280–300 mOsmol). To record spontaneous excitatory postsynaptic currents (sEPSCs), GABA_A_-mediated neurotransmission was blocked with 50 µM picrotoxin (Tocris, R & D Systems; Minneapolis, MN). Miniature excitatory postsynaptic currents (mEPSCs) were recorded in the presence of 1 µM tetrodotoxin (to block the action potentials) and 50 µM picrotoxin to block inhibitory neurotransmission (Tocris, R & D Systems; Minneapolis, MN) in the external solution, and 10 µM QX-314 (Tocris, R & D Systems; Minneapolis, MN) in the internal solution, as previously described^85, 86^. The membrane potential was held at −70 mV throughout all experiments. Data were amplified and filtered at 2 kHz by a patch-clamp amplifier (Multiclamp 700B), digitalized (DIGIDATA 1440A), stored, and analyzed by pCLAMP (Molecular Devices; Union City, CA). Data were discarded when the input resistance changed >20% during recording.

### Statistics

Immunofluorescence and electrophysiological data were analyzed for changes in treatment conditions when compared to control cells treated with vehicle. Data were analyzed with ANOVA followed by Tukey-Kramer’s multiple comparisons test using InStat and Prism software (GraphPad). Electrophysiology data were analyzed by calculating the frequency of events from at least 5 neurons per treatment condition per neuronal culture, from at least 3 cultures. *p* values < 0.05 were considered significant.

## Electronic supplementary material

Supplementary Information
