# Supplementary material for: Role of Akt-independent mTORC1 and GSK3β signaling in sublethal NMDA-induced injury and the recovery of neuronal electrophysiology and survival
Source: Sci Rep. 2017 May 8;7:1539. doi: 10.1038/s41598-017-01826-w (PMC5431483; doi:10.1038/s41598-017-01826-w)
Supplement: Supplementary file 1 — Supplementary Information [file 41598_2017_1826_MOESM1_ESM.pdf]

Role of Akt-independent mTORC1 and GSK3β signaling in sublethal NMDA-induced injury and the recovery of neuronal electrophysiology and survival

Przemyslaw Swiatkowski[1,2], Ina Nikolaeva[1,2], Gaurav Kumar[1], Avery Zucco[1,3], Barbara F. Akum[1], Mihir V. Patel[1,3], Gabriella D'Arcangelo[1], and Bonnie L. Firestein[1,*]

[1]Department of Cell Biology and Neuroscience, [2]Graduate Program in Molecular Biosciences, and [3]Graduate Program in Neurosciences, 604 Allison Road, Rutgers University, Piscataway, New Jersey 08854-8082

*Address correspondence to Dr. Bonnie L. Firestein (firestein@biology.rutgers.edu); phone: 848-445-8045; fax: 732-445-5870.

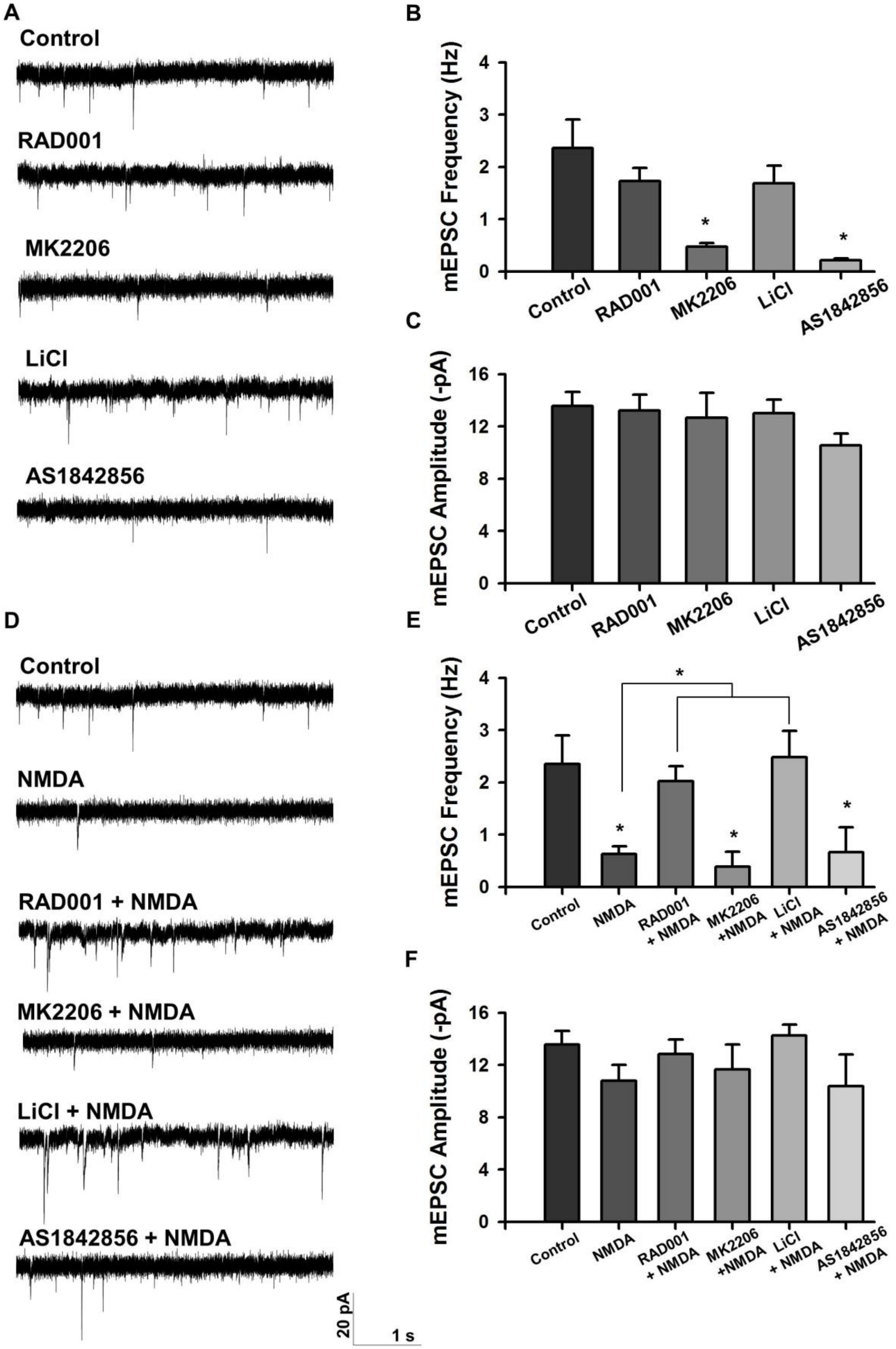
A
Control
RAD001
MK2206
LiCl
AS1842856
B
mEPSC Frequency (Hz)
*
*
Control
RAD001
MK2206
LiCl
AS1842856
C
mEPSC Amplitude (-pA)
D
Control
NMDA
RAD001 + NMDA
MK2206 + NMDA
LiCl + NMDA
AS1842856 + NMDA
20 pA
1 s
E
F
+ NMDA
+NMDA

Supplementary Figure S1. Inhibition of mTOR and GSK3β lead to improved frequency of mEPSCs 2 hours following NMDA-induced injury. *A,D*. Representative traces of mEPSCs recorded from rat cortical neurons treated with <0.1% DMSO (control; n=12), 20µM NMDA (n=10), 5 µM RAD001 (n=12), 2 µM MK2206 (n=7), 10mM LiCl (n=7), 1 µM AS1842856 (n=7), RAD + NMDA (n=16), MK2206 + NMDA (n=7), LiCl + NMDA (n=12), and AS1842856 + NMDA (n=6). *B-C*. Bar graph analysis of sEPSC frequency and amplitude following 4 hour baseline drug treatments and 2 hour recovery period. *E-F*. Bar graph analysis of sEPSC frequency and amplitude following 4 hour drug treatment, 5 minute 20µM NMDA-induced injury, and two hour recovery period. mEPSC data suggest that deficits in neuronal electrophysiology at two hours following injury are independent of neuronal excitability. *$p<0.05$ determined by one-way ANOVA followed by Tukey-Kramer multiple comparisons test. Error bars indicate ± SEM.

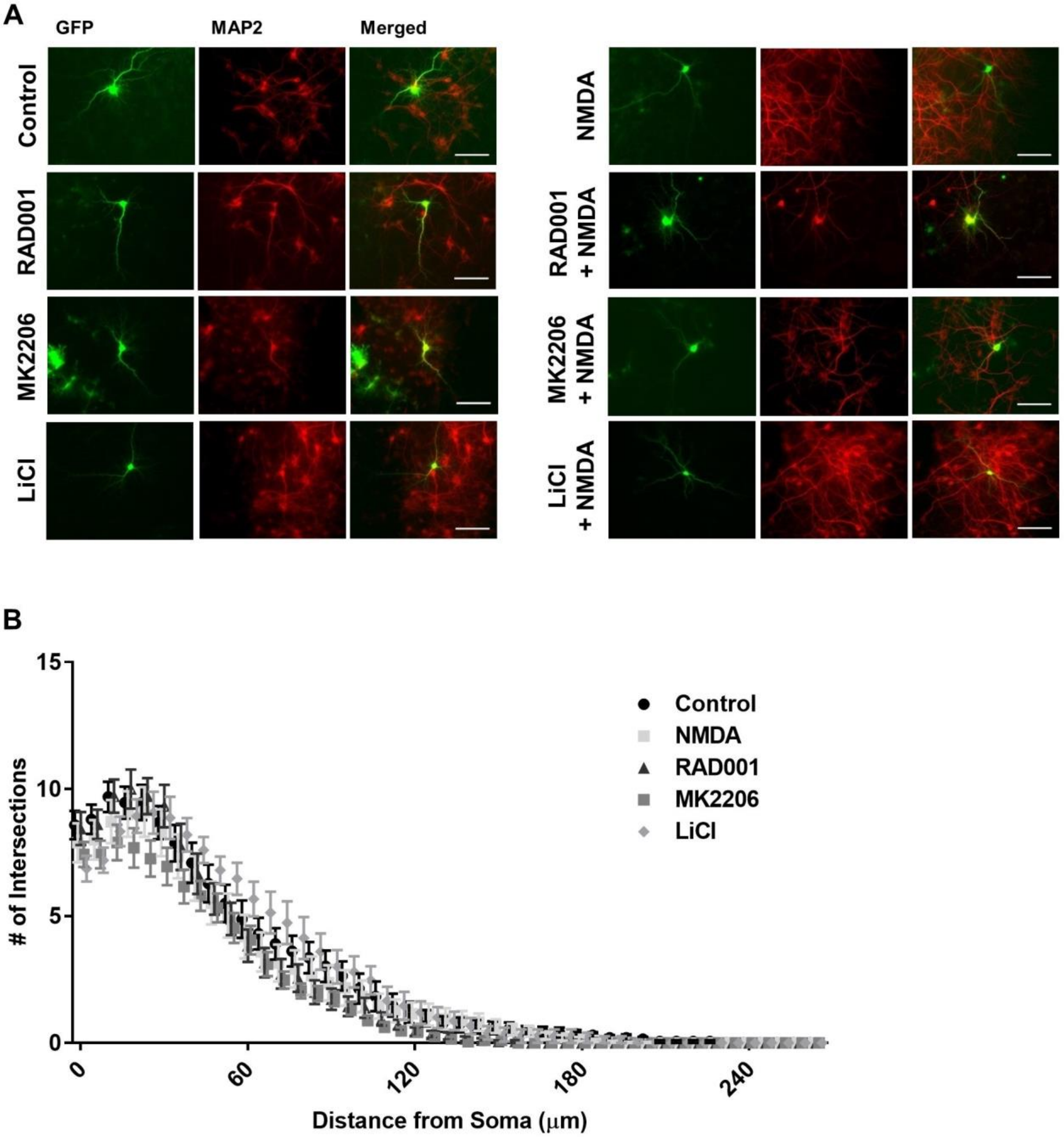

Supplementary Figure S2. PI3K/Akt/mTOR pathway inhibitors do not affect dendritic arborization. Primary hippocampal neurons were transfected with cDNA encoding GFP on DIV 14. On DIV 20, neurons were treated with 0.1% DMSO (control), NMDA, RAD001, MK2206, LiCl, then medium with or without 20 μM NMDA. *A.* Representative images showing GFP fluorescence and neuron-specific MAP2 immunostaining. Scale bar: 100μm. *B.* Sholl analysis

reveas no differences between treatment groups in number of intersections of dendrites. Each data set represents 20 neurons from three different trials. No difference between any condition and control was observed. *p* value determined by one-way ANOVA followed by Tukey-Kramer multiple comparisons test. Error bars indicate ± SEM.

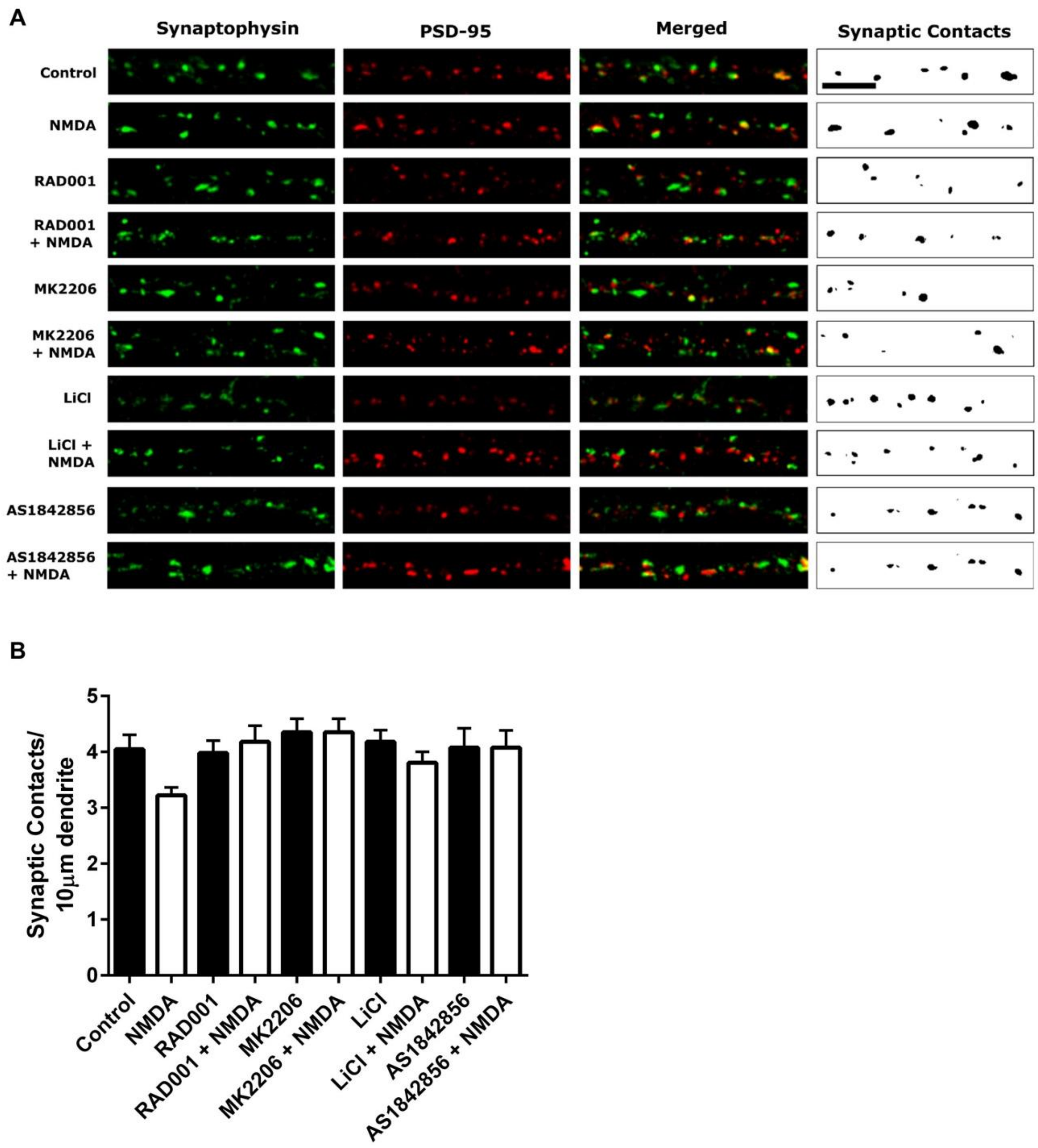

Supplementary Figure S3. PI3K/Akt/mTOR pathway inhibitors do not affect synaptic clusters. On DIV 14, neurons were treated with 0.1% DMSO (control), 5 μM RAD001, 2 μM MK2206, or 10 mM LiCl, with or without 20 μM NMDA. A. Representative fluorescence images showing excitatory synapses with immunostaining for PSD-95 on dendrites and synaptophysin on axons. Merged panels show apposition of PSD-95 and synaptophysin immunostaining, and hence

synaptic clusters, in yellow. Apposed clusters (excitatory synapses) from merged panels are shown as binary images. B. Quantitation of excitatory synapses counted from 15-30 µm dendrite lengths. Each data set represents 20 neurons from three different trials. No difference between any condition and control was observed. *p* value determined by one-way ANOVA followed by Tukey's multiple comparisons test. Error bars indicate ± SEM. Scale bar= 5 µm.
